# Butylidenephthalide Blocks Potassium Channels and Enhances Basal Tension in Isolated Guinea-Pig Trachea

**DOI:** 10.1155/2014/875230

**Published:** 2014-07-10

**Authors:** Hsin-Te Hsu, You-Lan Yang, Wan-Chen Chen, Chi-Ming Chen, Wun-Chang Ko

**Affiliations:** ^1^Department of Otolaryngology, Taipei Medical University Hospital, 252 Wu-Hsing Street, Taipei 110, Taiwan; ^2^School of Respiratory Therapy, College of Medicine, Taipei Medical University, Taiwan; ^3^Department of Pharmacology, College of Medicine, Taipei Medical University, 250 Wu-Hsing Street, Taipei 110, Taiwan; ^4^Department of Medicinal Chemistry, College of Pharmacy, Taipei Medical University, Taipei, Taiwan

## Abstract

Butylidenephthalide (Bdph, 30~300 *μ*M), a constituent of *Ligusticum chuanxiong* Hort., significantly enhanced tension in isolated guinea-pig trachea. In this study, we investigate the mechanism(s) of Bdph-induced contraction in the tissue. Isolated trachea was bathed in 5 mL of Krebs solution containing indomethacin (3 *μ*M), and its tension changes were isometrically recorded. Cromakalim (3 *μ*M), an ATP-dependent K^+^ channel opener, significantly antagonized the Bdph-induced enhancement of baseline tension. Bdph (300 *μ*M) also significantly antagonized cromakalim-induced relaxation. Bdph (300 *μ*M) did not significantly influence the antagonistic effects of glibenclamide (GBC, 1 *μ*M) and tetraethylammonium (TEA, 8 mM) against the cromakalim-induced relaxation. However, Bdph (300 *μ*M) and 4-aminopiridine (4-AP, 5 mM), a blocker of K_*v*_1 family of K^+^ channels, in combination significantly rightward shifted the log concentration-relaxation curve of cromakalim. The antagonistic effect of the combination almost equals the sum of the individual effects of Bdph and 4-AP, suggesting that the antagonistic mechanism of Bdph may be similar to that of 4-AP. All calcium channel blockers influenced neither the baseline tension nor antagonistic effect of Bdph against cromakalim. In conclusion, Bdph may be similar to 4-AP, a blocker of K_*v*_1 family of K^+^ channels, to enhance the baseline tension of guinea-pig trachea.

## 1. Introduction

The rhizomes of* Ligusticum chuanxiong* Hort. (previously named* L. wallichii* Franch.) and* Angelica sinensis* Diels. (Apiaceae) have been used by the Chinese for several thousand years. In ancient medical literature, such as Shen-Nung-Pen-Tsao-Ching, the rhizome of* L. chuanxiong* Hort. was delineated to prevent and restore stroke-induced dyskinesia. We reported that butylidenephthalide (Bdph), a neutral oil constituent of the rhizome, inhibited cyclooxygenase to have antiplatelet effects [1]. Other investigators reported that shimotsu-to, a prescription of traditional Chinese medicine (TCM), had antiproliferative effects in primary cultures of mouse aorta smooth muscle cells [2], mainly due to* Cnidium* rhizome-derived phthalides, such as senkyunolide, ligustilide, and Bdph [3]. Both antiplatelet and antiproliferative effects of these crude drugs benefit to prevent stroke. To recover from stroke-induced dyskinesia the damaged nerve cells need to be repaired mainly by themselves. The vasodilating effects of Bdph [4–6] improve the circulation and may partially benefit this restoration. Recently, Bdph was reported to provide neuroprotection by reducing the release of various proinflammatory molecules from activated microglia [7]. It is also reported to maintain stem cell pluripotency by activating the Jak2/Stat3 pathway and increasing the efficiency of induced pluripotent stem cells generation [8]. These results highlight the ability for these crude drugs to aid in the recovery from dyskinesia. Interestingly, Bdph was also reported to inhibit growth of malignant brain tumor [9], lung adenocarcinoma [10], and glioblastomas [11] with a high therapeutic ratio [12].

Bdph (50~250 *μ*M) was reported to noncompetitively inhibit ACh-, KCl-, and BaCl_2_-induced contractions in guinea-pig ileum [13]. Bdph (30~300 *μ*M) was also reported to noncompetitively inhibit phenylephrine- and KCl-induced contractions in rat aortic rings [6]. However, in preliminary test, we found that Bdph (30~300 *μ*M) failed to, except at very high concentrations (600~1000 *μ*M), inhibit histamine (10 *μ*M)-induced contraction in isolated guinea-pig trachea. More surprisingly, we found that Bdph (30~300 *μ*M) alone can significantly induce enhancement of baseline tension in the tissue. Therefore we are interested in investigating the mechanism(s) of Bdph-induced contraction in the tissue.

## 2. Methods and Materials

### 2.1. Drugs and Animals

**Figure 1:**
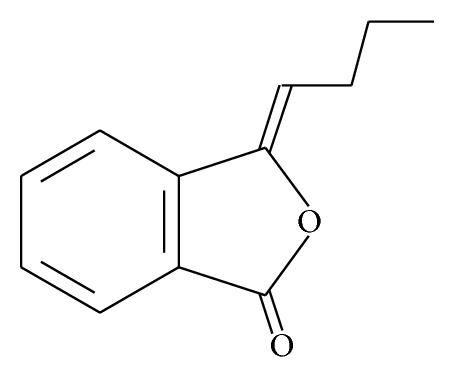
The chemical structure of butylidenephthalide (Bdph, mol. wt. 188.23).

Bdph was synthesized and yielded a light yellow oily substance according to the previously described method [14]. Its purity (99.8%) was analyzed by using high performance liquid chromatography and the structure is shown in Figure 1 [15]. 4-Aminopiridine (4-AP), atropine sulfate, *α*-chymotrypsin, diltiazem, glibenclamide (GBC), histamine diphosphate, indomethacin, nicardipine, nifedipine (Nif), pyrilamine maleate, tetraethylammonium bromide (TEA), and verapamil (Vrp) were purchased from Sigma-Aldrich, St. Louis, MO. U.S.A. Papaverine was purchased from Narcotics Bureau, Taipei, Taiwan. Cromakalim, methysergide, and FPL 557121 were gifts from SmithKline Beecham Pharmaceutical, U.K., Sandoz, Swiss, and Fisons, U.K., respectively.

Male Hartley guinea-pigs (250~400 g) were obtained from the Animal Center of the National Science Council (Taipei, Taiwan). The animals were housed in ordinary cages at 22 ± 1°C with a humidity of 50%~60% under a constant 12/12-h light/dark cycle and provided with food and water* ad libitum*. Under a protocol approved by the Animal Care and Use Committee of Taipei Medical University, the following* in vitro* experiments were performed.

### 2.2. Tracheal Preparation

The guinea-pigs were sacrificed by cervical dislocation after anesthesia, and their tracheas were removed. Each trachea was cut into six segments. Each segment consisted of three cartilage rings. All segments were cut open opposite the trachealis. The segments were randomized to minimize regional variability. Each segment was tied at one end to a holder via silk sutures, placed in 5 mL of Krebs solution containing indomethacin (3 *μ*M) throughout the entire experiment, bubbled with a 95% O_2_ and 5% CO_2_ mixture at 37°C, and attached by the other end to a force displacement transducer (Grass FT03) for the isometric recording of tension changes on a polygraph (Gould RS3200). The composition of the Krebs solution was (mM): NaCl 120, KCl 4.7, MgSO_4_ 0.5, KH_2_PO_4_ 1.2, CaCl_2_ 2.5, NaHCO_3_ 25, and dextrose 11.0. The tissues were suspended under an initial tension of 1.5 g and allowed to equilibrate for 1 h with washing at 15-min intervals. After equilibration, the following experiments were performed.

### 2.3. Bdph Enhanced Baseline Tension but Relaxed Precontraction at High Concentrations

Bdph (30~1000 *μ*M) or its vehicle (0.03~1% ethyl alcohol) was cumulatively added to examine the tension change of baseline. After the histamine (10 *μ*M)-induced precontraction reached steady state, Bdph (30~1000 *μ*M) or its vehicle was cumulatively added to the organ bath. At the end of experiment, papaverine (0.1 mM) was added to maximally relax the tissue and to standardize the relaxation (100%). The log concentration-response curves of Bdph for both baseline tension and relaxation were constructed.

### 2.4. Cromakalim Antagonized Bdph-Induced Enhancement of Baseline Tension

In order to examine the possible transmitter(s) or mediators which may enhance the tracheal baseline tension, some pharmacological agents, such as 1 *μ*M atropine (a cholinergic antagonist), 1 *μ*M FPL 55712 (a leukotriene receptor antagonist) [16], 1~10 *μ*M pyrilamine (a histamine receptor antagonist), 1 *μ*M methysergide (a serotonin receptor antagonist), and 2 u/mL *α*-chymotrypsin (a neuropeptidase), were added 30 min prior to the cumulative addition of Bdph (30~300 *μ*M). However, 1~3 *μ*M cromakalim (an ATP-sensitive K^+^ channel opener) was pretreated for only 10 min which was enough to reach equilibration [17].

### 2.5. Bdph Also Antagonized Cromakalim-Induced Relaxation

After preincubation of Bdph (30~300 *μ*M) or its vehicle for 20 min, histamine (10~30 *μ*M) was added to reach a half-maximal contraction and then cumulatively added cromakalim (0.1~10 *μ*M). At the end of experiment, papaverine (0.1 mM) was added to maximally relax the tissue and to standardize the relaxation (100%). The log concentration-response curves of cromakalim in the absence and presence of Bdph were constructed.

### 2.6. Interaction between Bdph and Other K^+^ Channel Blockers to Antagonize Cromakalim-Induced Relaxation

After preincubation of Bdph (100 or 300 *μ*M) and other K^+^ channel blockers, such as GBC (1 *μ*M), TEA (8 mM), and 4-AP (5 mM) alone or combination for 20 min, histamine (10~30 *μ*M) was added to reach a half-maximal contraction, and then cromakalim (0.1~10 *μ*M) was cumulatively added. At the end of experiment, papaverine (0.1 mM) was added to maximally relax the tissue and to standardize the relaxation (100%). The log concentration-response curves of cromakalim in the absence and presence of drug(s), such as Bdph and other K^+^ channel blockers, alone or combination, were constructed.

### 2.7. Interaction between Bdph and Other Ca^2+^ Channel Blockers to Antagonize Cromakalim-Induced Relaxation

First, Vrp, Nif, diltiazem, nicardipine, or their vehicles were cumulatively added to examine the tension change of baseline in the isolated trachea. Second, after preincubation of Bdph (300 *μ*M) or its vehicle for 20 min, histamine (10~30 *μ*M) was added to reach a half-maximal contraction, and then Vrp (0.01~10 *μ*M) or Nif (0.001~1 *μ*M) was cumulatively added. At the end of experiment, papaverine (0.1 mM) was added to maximally relax the tissue and to standardize the relaxation (100%). The log concentration-response curves of Vrp and Nif in the absence and presence of Bdph were constructed. Third, after preincubation of Bdph (300 *μ*M), Vrp (1 *μ*M), and Nif (0.1 *μ*M) alone or combination for 20 min, histamine (10~30 *μ*M) was added to reach a half-maximal contraction, and then cromakalim (0.1~10 *μ*M) was cumulatively added. At the end of experiment, papaverine (0.1 mM) was added to maximally relax the tissue and to standardize the relaxation (100%). The log concentration-response curves of cromakalim in the absence and presence of drugs, such as Bdph, Vrp, and Nif, alone or combination were constructed.

### 2.8. Statistical Analysis

The tracheal contraction was expressed as percentage of maximal contraction (100%), with some exceptions expressed as tension. However, the tracheal relaxation was expressed as percentage of maximal relaxation induced by papaverine (100%) at the end of experiment. All values are expressed as mean ± SEM, *n* is the number of experiment. Student's unpaired *t*-test was used for statistical analysis between test and control with *P* values < 0.05 being regarded as significant.

## 3. Results

### 3.1. Effects of Bdph on Baseline and Histamine-Induced Precontraction

**Figure 2:**
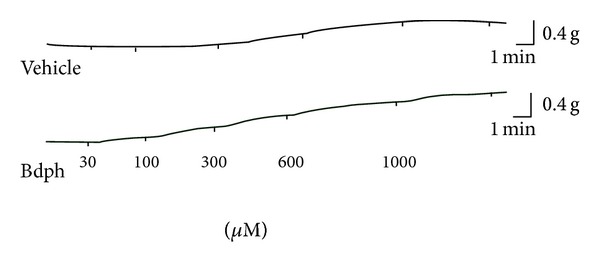
Typical tracing of baseline tension change induced by cumulative butylidenephthalide (Bdph, lower panel) compared to its vehicle (0.03~1% ethyl alcohol, upper panel) in isolated guinea-pig trachea. The vertical line indicates tension change.

**Figure 3:**
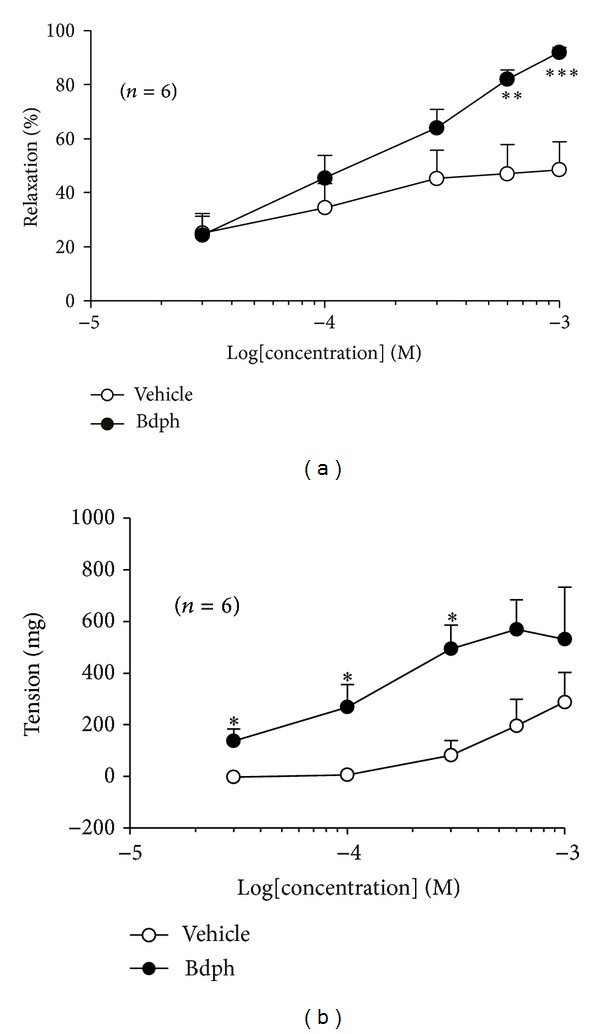
Log concentration relaxant (a) and contractile (b) response curves of butylidenephthalide (Bdph) in isolated guinea-pig trachea. The relaxant and contractile effects of cumulative Bdph (30~1000 *μ*M) on histamine (10 *μ*M)-induced precontraction and on baseline tension were performed as method described, respectively. All values are shown as mean ± SEM, and *n* is the number of experiments. *P < 0.05, **P < 0.01, and ***P < 0.001 compared to its vehicle.

The effect of Bdph (30~1000 *μ*M), compared to its vehicle, on the baseline tension in isolated guinea-pig trachea is shown in Figures 2 and 3(b). Bdph (30~300 *μ*M) did not significantly relax the histamine (10 *μ*M)-induced precontraction, except at higher concentrations of 600~1000 *μ*M in the tissue (Figure 3(a)). In contrast, Bdph (30~300 *μ*M) significantly enhanced its baseline tension (Figure 3(b)).

### 3.2. Cromakalim Antagonized Bdph-Induced Enhancement of Baseline Tension

**Figure 4:**
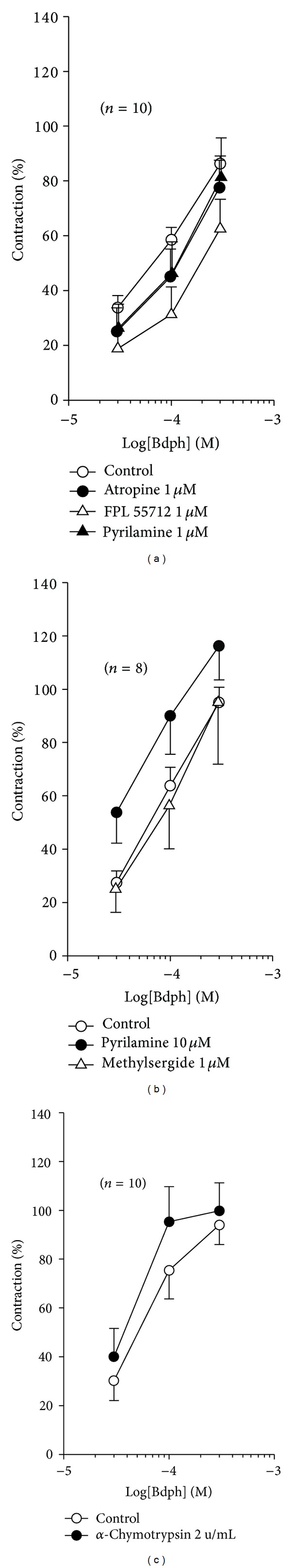
Inhibitory effects of atropine, FPL 55712, or pyrilamine 1 *μ*M (a), as well as pyrilamine 10 *μ*M or methysergide (b) and *α*-chymotrypsin (c) on cumulative butylidenephthalide- (Bdph-) induced contraction of baseline tension in isolated guinea-pig trachea. All values are shown as mean ± SEM, and *n* is the number of experiments. There is no significant difference between test and respective control.

**Figure 5:**
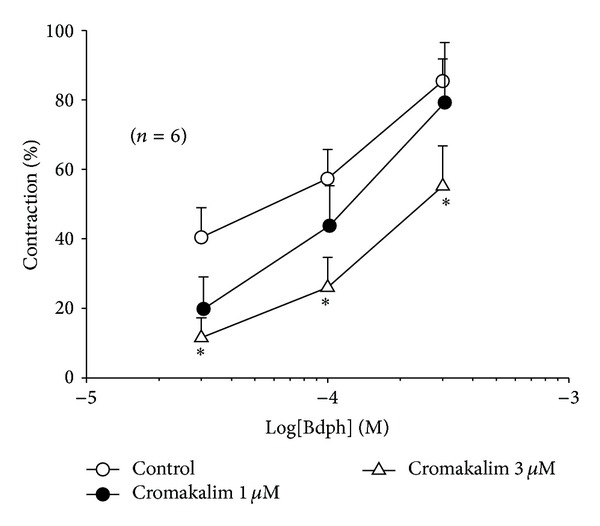
Inhibitory effect of cromakalim on cumulative butylidenephthalide (Bdph)-induced contraction of baseline tension in isolated guinea-pig trachea. All values are shown as mean ± SEM, and *n* is the number of experiments. *P < 0.05 compared to its vehicle.

Atropine (1 *μ*M), FPL 55712 (1 *μ*M), pyrilamine (1 and 10 *μ*M), methysergide (1 *μ*M), or *α*-chymotrypsin (2 u/mL) did not significantly influence the Bdph-induced enhancement of baseline tension (Figure 4). However, cromakalim (3 *μ*M) significantly antagonized the Bdph-induced enhancement of baseline tension (Figure 5).

### 3.3. Bdph Also Antagonized Cromakalim-Induced Relaxation

**Figure 6:**
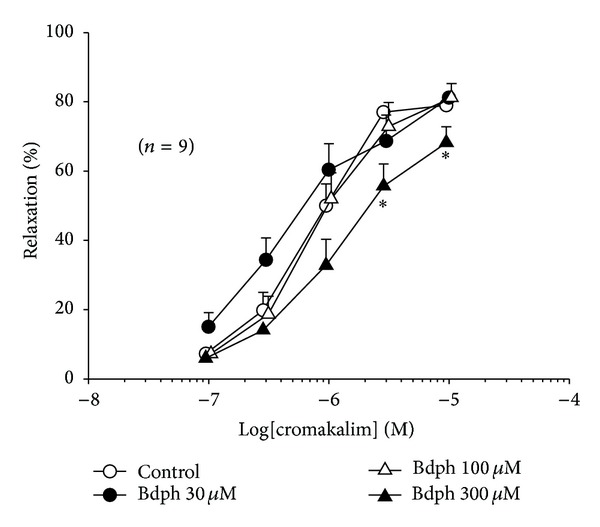
Inhibitory effect of butylidenephthalide (Bdph) on cumulative cromakalim-induced relaxant response to histamine-induced precontraction in isolated guinea-pig trachea. All values are shown as mean ± SEM, and *n* is the number of experiments. *P < 0.05 compared to its vehicle.

Bdph (300 *μ*M) significantly antagonized cromakalim-induced relaxation (Figure 6).

### 3.4. Interaction between Bdph and Other K^+^ Channel Blockers to Antagonize Cromakalim-Induced Relaxation

**Figure 7:**
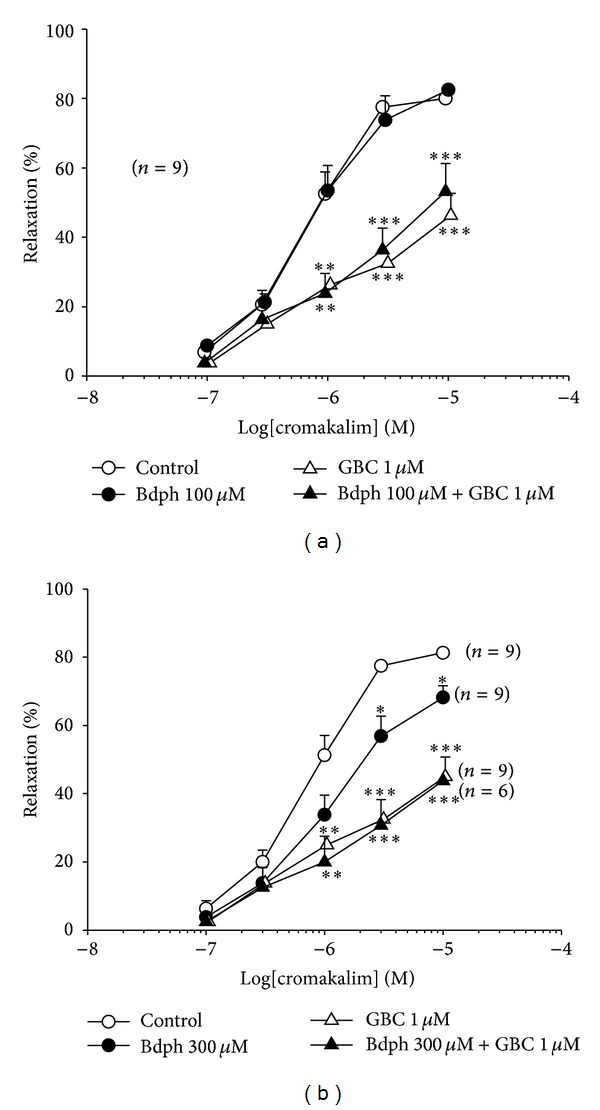
Influences of glibenclamide (GBC) to the antagonistic effects of butylidenephthalide (Bdph) 100 *μ*M (a) and 300 *μ*M (b) on cumulative cromakalim-induced relaxant response to histamine-induced precontraction in isolated guinea-pig trachea. All values are shown as mean ± SEM, and *n* is the number of experiments. *P < 0.05, **P < 0.01, and ***P < 0.001 compared to its vehicle.

**Figure 8:**
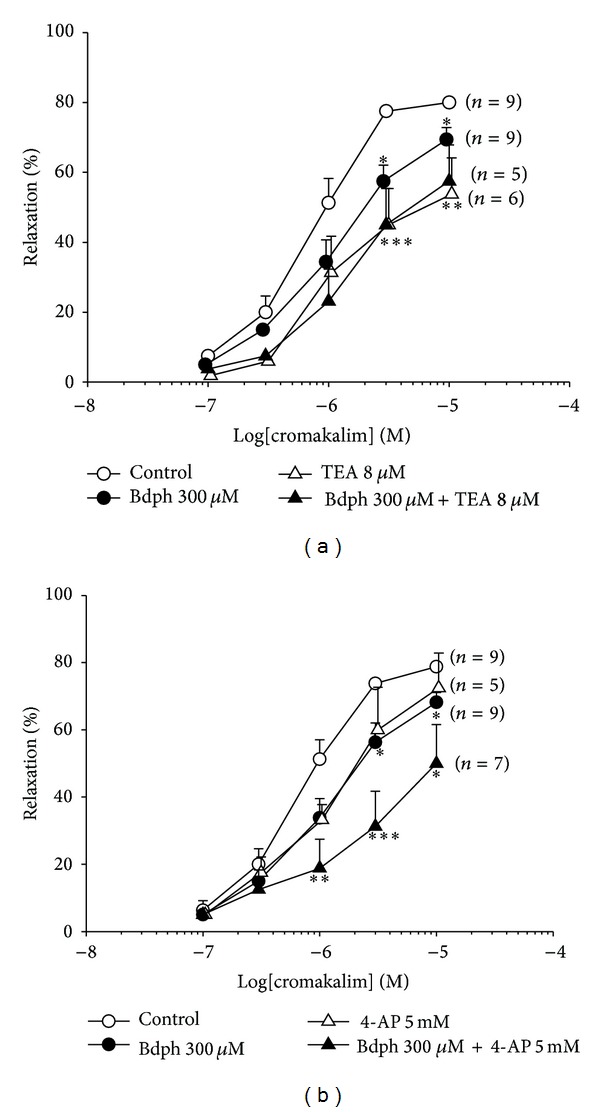
Influences of tetraethylamine (TEA, (a)) and 4-aminopyridine (4-AP, (b)) to the antagonistic effect of butylidenephthalide (Bdph) on cumulative cromakalim-induced relaxant response to histamine-induced precontraction in isolated guinea-pig trachea. All values are shown as mean ± SEM, and *n* is the number of experiments. *P < 0.05, **P < 0.01, and ***P < 0.001 compared to its vehicle.

Bdph at concentrations of 100 *μ*M and 300 *μ*M did not significantly influence the antagonistic effects of GBC (1 *μ*M) against the cromakalim-induced relaxation (Figure 7). Bdph (300 *μ*M) never influenced the antagonistic effects of TEA at 8 mM (Figure 8(a)). However, Bdph (300 *μ*M) and 4-AP (5 mM) in combination significantly antagonized the cromakalim-induced relaxation, compared to the individual effects on the relaxation, and rightward shifted the log concentration-response curve of cromakalim. The antagonistic effect of the combination is almost equal to the sum of individual effects (Figure 8(b)).

### 3.5. Interaction between Bdph and Other Ca^2+^ Channel Blockers to Antagonize Cromakalim-Induced Relaxation

**Figure 9:**
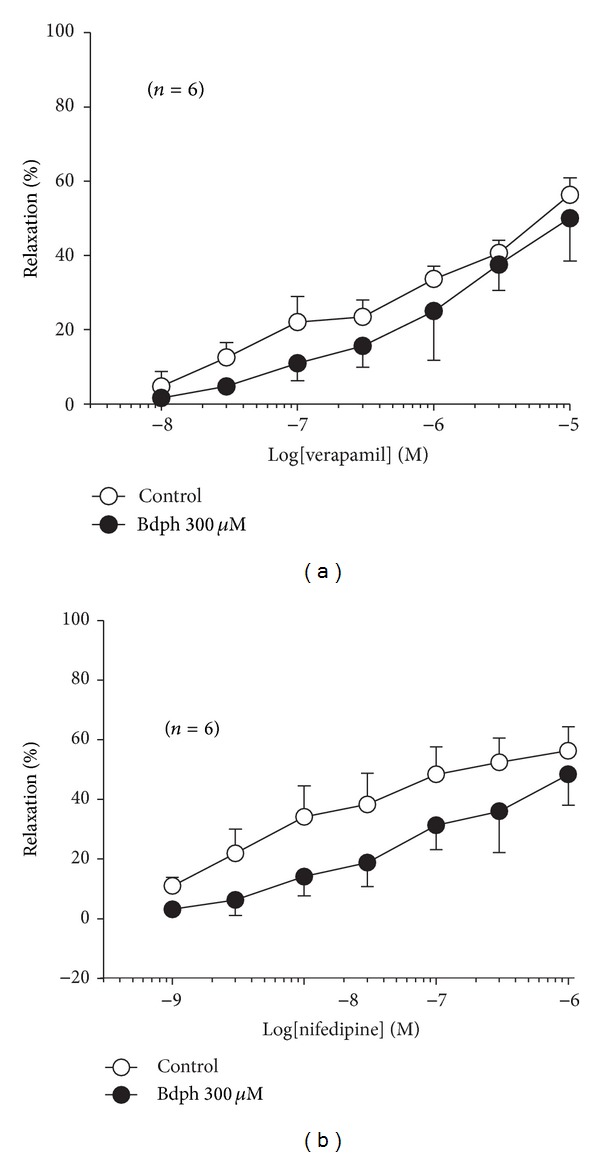
Antagonistic effects of butylidenephthalide (Bdph) on cumulative verapamil-induced (a) and nifedipine-induced (b) relaxant response to histamine-induced precontraction in isolated guinea-pig trachea. All values are shown as mean ± SEM, and *n* is the number of experiments. There is no significant difference between test and respective control.

**Figure 10:**
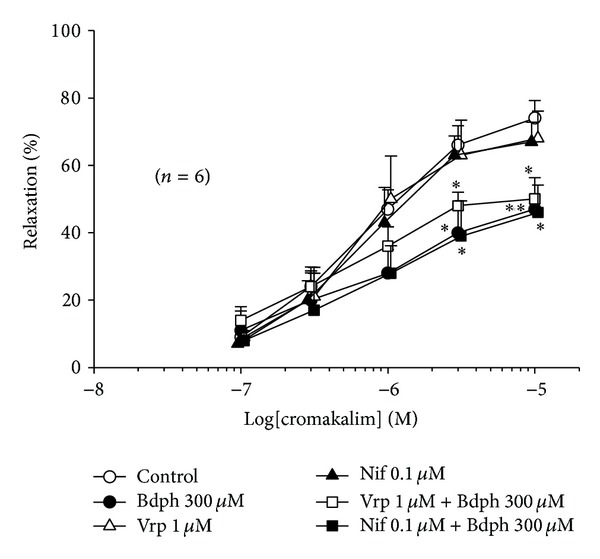
Influences of verapamil (Vrp) and nifedipine (Nif) to the antagonistic effect of butylidenephthalide (Bdph) on cumulative cromakalim-induced relaxant response to histamine-induced precontraction in isolated guinea-pig trachea. All values are shown as mean ± SEM, and *n* is the number of experiments. *P < 0.05 and **P < 0.01 compared to its vehicle.

All Ca^2+^ channel blockers used did not enhance or reduce the baseline tension of the isolated guinea-pig trachea (data not shown). Bdph did not influence the relaxant effects of Vrp (Figure 9(a)) and Nif (Figure 9(b)) on the histamine-induced precontraction. Vrp (1 *μ*M) and Nif (0.1 *μ*M) also did not influence the antagonistic effect of Bdph (300 *μ*M) against the cromakalim-induced relaxation (Figure 10).

## 4. Discussion

The present results suggest that the enhancement of basal tension by Bdph is unrelated to the release of cholinergic transmitter, leukotrienes, histamine, serotonin, and neuropeptides [18]. It is also unrelated to the release of prostaglandins, as the experiment was conducted throughout in the presence of indomethacin. However, the enhancement was antagonized by cromakalim (3 *μ*M), an ATP-sensitive K^+^ channel opener [17], which may increase outflux of K^+^ and hyperpolarize the membrane of tracheal smooth muscle cells and cause relaxation. Furthermore, Bdph (300 *μ*M) also antagonized and rightward shifted the log concentration-relaxation curve of cromakalim on histamine-induced precontraction in the isolated guinea-pig trachea (Figure 6). Thus, Bdph may be a kind of K^+^ channels blockers, which have been reviewed to have a potential clinical use for Alzheimer disease [19]. Indeed, Bdph have been reported to reverse the deficits of inhibitory avoidance performance and improve memory in rats [20]. GBC (1 *μ*M), a specific ATP-sensitive K^+^ channel blocker [17], effectively antagonized and rightward shifted the curve of cromakalim. Bdph neither at 100 *μ*M nor at 300 *μ*M influenced the antagonistic effect of GBC. Also, Bdph at 300 *μ*M did not affect the antagonistic effect of TEA (8 mM), a nonselective big (BKca) and intermediate (IKca) conductance Ca^2+^-activated K^+^ channels blocker [21]. However, Bdph at 300 *μ*M significantly enhanced the antagonistic effect of 4-AP (5 mM) and rightward shifted the curve in the combination. The antagonistic effect of the combination was almost the sum of individual effects (Figure 8(b)). This result strongly suggests that the mechanism of Bdph may be similar to that of 4-AP to antagonize cromakalim. The mechanism of Bdph was unrelated to Ca^2+^-dependent K^+^ channels, as all Ca^2+^ channel blockers did not influence the antagonistic effect of Bdph against cromakalim.

Episodic ataxia type 2 (EA2) is a form of hereditary neurological disorder caused by cerebellar malfunction and is characterized by interictal ataxia and frequent attacks of dyskinesia, vertigo, and imbalance [22]. Recently, 4-AP was reported to treat EA2 [23, 24]. The targets of 4-AP are K_*v*_1 family of K^+^ channels, possibly the K_*v*_1.5 subtype [25]. Further investigation is needed to determine whether Bdph is useful in treating EA2.

In conclusion, Bdph (30~300 *μ*M) concentration-dependently evoked an enhancement of baseline tension in isolated guinea-pig trachea. The enhancement was antagonized by cromakalim, and Bdph (300 *μ*M) also antagonized cromakalim-induced relaxation. Furthermore, Bdph (300 *μ*M) and 4-AP (5 mM) in combination rightward shifted the log concentration-response curve of cromakalim and significantly antagonized the cromakalim-induced relaxation. The antagonistic effect of the combination is almost equal to the sum of individual effects. Therefore, Bdph may be similar to 4-AP, a blocker of K_*v*_1 family of K^+^ channels, to enhance the baseline tension of guinea-pig trachea.

## References

[1] C. M. Teng (1987). Antiplatelet effect of butylidenephthalide. *Biochimica et Biophysica Acta*.

[2] Kobayashi S, Mimura Y, Notoya K, Kimura I, Kimura M (1992). Antiproliferative effects of the traditional Chinese medicine Shimotsu-to, its component cnidium rhizome and derived compounds on primary cultures of mouse aorta smooth muscle cells. *Japanese Journal of Pharmacology*.

[3] Kobayashi S, Mimura Y, Naitoh T, Kimura I, Kimura M (1993). Chemical structure-activity of cnidium rhizome-derived phthalides for the competence inhibition of proliferation in primary cultures of mouse aorta smooth muscle cells. *Japanese Journal of Pharmacology*.

[4] Ko WC, Chang LD, Wang GY, Lin LC (1994). Pharmacological effects of butylidenephthalide. *Phytotherapy Research*.

[5] Ko WC, Sheu JR, Tzeng SH, Chen CM (1998). The selective antianginal effect without changing blood pressure of butylidenephthalide in conscious rats. *Planta Medica*.

[6] Ko W-C, Charng C-Y, Sheu J-R, Tzeng S-H, Chen C-M (1998). Effect of butylidenephthalide on calcium mobilization in isolated rat aorta. *Journal of Pharmacy and Pharmacology*.

[7] Nam KN, Kim KP, Cho KH (2013). Prevention of inflammation-mediated neurotoxicity by butylidenephthalide and its role in microglial activation. *Cell Biochemistry and Function*.

[8] Liu S, Harn H, Chien Y (2012). n-Butylidenephthalide (BP) maintains stem cell pluripotency by activating Jak2/Stat3 pathway and increases the efficiency of iPS cells generation. *PLoS ONE*.

[9] Tsai NM, Chen YL, Lee CC (2006). The natural compound *n*-butylidenephthalide derived from Angelica sinensis inhibits malignant brain tumor growth in vitro and in vivo. *Journal of Neurochemistry*.

[10] Wei C, Lin C, Yu Y (2009). N-Butylidenephthalide induced apoptosis in the A549 human lung adenocarcinoma cell line by coupled down-regulation of AP-2*α* and telomerase activity. *Acta Pharmacologica Sinica*.

[11] Lin PC, Lin SZ, Chen YL (2011). Butylidenephthalide suppresses human telomerase reverse transcriptase (TERT) in human glioblastomas. *Annals of Surgical Oncology*.

[12] Zhang H, Han T, Yu CH (2012). Analysis of the chemical composition, acute toxicity and skin sensitivity of essential oil from rhizomes of Ligusticum chuanxiong. *Journal of Ethnopharmacology*.

[13] Ko WC (1980). A newly isolated antispasmodic—butylidenephthalide. *Japanese Journal of Pharmacology*.

[14] Mowry DT, Ringwald EL, Renoll M (1949). Vinyl aromatic compounds. VI. Alkylidenephthalides and related compounds. *Journal of the American Chemical Society*.

[15] Lin LC, Wang CB, Koh VC, Ko WC (1984). Synthesis, properties, and molecular structure of alkylidenephthalides. *Bulletin of the Institute of Chemistry Academia Sinica*.

[16] Parisi VM, Phernetton TM, Rankin JHG (1985). Placental vascular responses to leukotriene receptor antagonist FPL 55712. *Prostaglandins*.

[17] Escande D, Thuringer D, Leguern S, Cavero I (1988). The potassium channel opener cromakalim (BRL 34915) activates ATP-dependent K^+^ channels in isolated cardiac myocytes. *Biochemical and Biophysical Research Communications*.

[18] Ko WC, Wang HL, Lei CB, Shih CH, Chung MI, Lin CN (2002). Mechanisms of relaxant action of 3-*O*-methylquercetin in isolated guinea pig trachea. *Planta Medica*.

[19] Lavretsky EP, Jarvik LF (1992). A group of potassium-channel blockers-acetylcholine releasers: new potentials for Alzheimer disease? A review. *Journal of Clinical Psychopharmacology*.

[20] Hsieh M-T, Wu C-R, Lin L-W, Hsieh C-C, Tsai C-H (2001). Reversal caused by n-butylidenephthalide from the deficits of inhibitory avoidance performance in rats. *Planta Medica*.

[21] Koslov DS, Andersson KE (2013). Physiological and pharmacological aspects of the vas deferens-an update. *Frontiers in Pharmacology*.

[22] Jen JC, Graves TD, Hess EJ, Hanna MG, Griggs RC, Baloh RW (2007). Primary episodic ataxias: diagnosis, pathogenesis and treatment. *Brain*.

[23] Strupp M, Brandt T (2006). Pharmacological advances in the treatment of neuro-otological and eye movement disorders. *Current Opinion in Neurology*.

[24] Strupp M, Kalla R, Glasauer S (2008). Aminopyridines for the treatment of cerebellar and ocular motor disorders. *Progress in Brain Research*.

[25] Alviña K, Khodakhah K (2010). The therapeutic mode of action of 4-aminopyridine in cerebellar ataxia. *Journal of Neuroscience*.

